# Supplementary material for: A UMLS-based spell checker for natural language processing in vaccine safety
Source: BMC Med Inform Decis Mak. 2007 Feb 12;7:3. doi: 10.1186/1472-6947-7-3 (PMC1805499; doi:10.1186/1472-6947-7-3)
Supplement: Additional file 13 — RAP application directory. Sets up directory for RAP (RDF application for PHP) source code [file 1472-6947-7-3-S13.gz › rap/doc/phpdoc/dataset/TriXSerializer.html]

xml version="1.0" encoding="iso-8859-1"?


Docs For Class TriXSerializer


## Class TriXSerializer

Description

Description |
Methods (details)

Temporary implementation of a TriX-Serializer

History:

- access: public
- author: Daniel Westphal (http://www.d-westphal.de)
- version: V0.9

Located in /syntax/TriXSerializer.php (line 19)

Method Summary

Description |
Methods (details)

TriXSerializer
TriXSerializer
(GraphSet &$graphSet)

void
serializeToFile
(string $fileName)

string
&serializeToString
()

Methods

Description |
Methods (details)

Constructor TriXSerializer (line 38)

Constructor Needs a reference to a graphSet

- access: public

TriXSerializer

TriXSerializer
(GraphSet &$graphSet)

- GraphSet
  &$graphSet

serializeToFile (line 60)

Serialize the dataset to a TriX string and save in file

- access: public

void

serializeToFile
(string $fileName)

- string
  $fileName

serializeToString (line 49)

Serialize the dataset to a TriX string

- access: public

string

&serializeToString
()

Documentation generated on Thu, 7 Jul 2005 13:44:01 +0200 by phpDocumentor 1.3.0RC3
